# Readability of online e-cigarette cessation information

**DOI:** 10.18332/tid/149906

**Published:** 2022-06-08

**Authors:** Lindsey A. Wood, Osayande Agbonlahor, Madeline M. Tomlinson, Savanna Kerstiens, Kolbie Vincent, Alison C. McLeish, Kandi L. Walker, Joy L. Hart

**Affiliations:** 1 Department of Communication, University of Louisville, Louisville, United States; 2 Christina Lee Brown Envirome Institute, University of Louisville, Louisville, United States; 3 Department of Psychological and Brain Sciences, University of Louisville, Louisville, United States; 4 American Heart Association Tobacco Center for Regulatory Science, Dallas, United States

**Keywords:** cessation, e-cigarettes, internet, online health information, readability

## Abstract

**INTRODUCTION:**

Given the growing awareness of the health risks associated with e-cigarettes, many users will access information about how to effectively quit using e-cigarettes, and the internet likely will be a popular source of information. However, little is known about the readability of online e-cigarette cessation information. Therefore, the goal of the current study was to assess the readability of webpage information about e-cigarette cessation.

**METHODS:**

A search of webpages was conducted using the following search terms: vaping addiction, quit vaping, quit Juul, stop vaping, stop Juul, and vaping cessation. The 464 webpages identified were coded for target audience, message valence, and source, and then assessed for reading level with the Flesch-Kincaid Grade Level assessment.

**RESULTS:**

On average, webpage content was written at a 7th grade reading level (Flesch-Kincaid Grade Level Mean=7.34, SD=2.22) and less than 25% of webpages met readability guidelines (i.e. ≤6th grade reading level). There were no differences in readability by target audience, message valence, or webpage source.

**CONCLUSIONS:**

The results suggest that most online content related to e-cigarette cessation is not written at a level that is easily understood by general audiences. Thus, tobacco control advocates should assess the reading level of their messaging to ensure wide accessibility of information.

## INTRODUCTION

Since the introduction of e-cigarettes in the US marketplace in 2006, their use has increased significantly in both youth and adults^1^. Recent estimates indicate that approximately 20% of youth and 5% of adults have used e-cigarettes in the past 30 days^2^. E-cigarettes are viewed by many as a healthier alternative to combustible cigarettes. Indeed, they may hold promise as an approach to harm reduction or a combustible cigarette cessation strategy^3^. However, their use is not without risk. For example, e-cigarette use is associated with increased risk for cardiovascular, immunologic, and respiratory diseases^4-6^. There are also significant associations between e-cigarette use and psychopathology, including major depressive disorder, generalized anxiety disorder, posttraumatic stress disorder, and attention deficit hyperactivity disorder^7-11^.

Given the growing body of evidence that e-cigarette use is associated with several adverse health outcomes, it is perhaps not surprising that, among a nationally-representative sample of current adult e-cigarette users in the US, over 60% reported wanting to quit in the next year^3^. Similarly, Amato et al.^12^ found that over 50% of youth enrolled in a vaping cessation program cited health as the top reason for wanting to quit. Toward this goal, many e-cigarette users will access information about how to effectively quit using e-cigarettes, and the internet likely will be a popular source of information^13^. To be beneficial, this health information must be easy to comprehend.

### Readability of online health information

One way to evaluate whether information can be easily understood is to examine its readability or reading level. In a recent systematic review, Daraz et al.^14^ concluded that the average readability of general online health information in North America was at the 10th to 15th (i.e. college) grade level. Studies of online cigarette smoking cessation content have found that under 10% of webpages were written at a 6th grade reading level^15^, and a quarter were written above a 12th grade reading level^16^. Although the average US adult reads at a 7th grade level^17^, nearly 21% – over a fifth of the adult population – read at or below a 5th grade level^18^. The reading levels of much online content far exceed the American Medical Association’s (AMA) recommendation to create written content for adults at no higher than a 6th grade reading level^19^.

The few studies that have examined the readability of online e-cigarette information found similar results. Wodi et al.^20^ found that only 24% of online e-cigarette safety information was written at or below a 6th grade level. Similarly, Park et al.^21^ concluded that, on average, text on e-cigarette-related webpages was written at the 10th to 14th grade level and that webpages promoting e-cigarette use were easier to read than webpages warning of e-cigarette dangers^21^. Further, in a study of the readability of online COVID-19 health information, Worrall et al.^22^ reported that webpages created by public health and government organizations were easier to read than those created by digital media ones. These findings suggest that there may be important differences in readability based on the target audience, message valence (positive or negative), and source; however, no such investigation has been done.

Extant research indicates that most online health information is written at a level that is difficult for many individuals to understand. However, there has been little examination of the readability of online e-cigarette content, particularly content related to cessation. Further, the Park et al.^21^ and Worrall et al.^22^ findings suggest that, in addition to examining overall readability, a more fine-grained analysis of webpages based on target audience, message valence, and source is needed. Therefore, the aim of the current study was to assess the reading level of online information related to e-cigarette cessation. A secondary goal was to examine possible differences in the readability of online text targeted to specific audiences (i.e. teens vs parents, users vs non-users), the valence of the e-cigarette information, and the source of the material.

## METHODS

A web search was conducted between 14 and 30 January 2021 using the following search terms: vaping addiction, quit vaping, quit Juul, stop vaping, stop Juul, and vaping cessation. These terms were chosen to approximate what individuals might search for online if they were interested in quitting their use of e-cigarettes. Given its market dominance, Juul was both a popular brand and a synonym for vaping; thus, Juul was included as a search term. Three coders conducted independent searches for each search term using the Google search engine. The Google Chrome Incognito browser was used to reduce the possibility that search results would be influenced by search history.

The first 100 webpages for each search term were recorded by three coders (N=1800; 100 webpages × six search terms × three coders) and then categorized by target audience (i.e. teens only, parents only, users only, and non-users only; all coded as yes/no), message valence (i.e. whether the webpage content included positive vaping messages; yes/no), and webpage source. As some webpages did not have a clear target audience, they were coded as ‘no’ for both audience options (i.e. teens and parents or users and non-users). Similar to previous research^22^, sources were coded as: 1) educational or scientific institutions (e.g. universities, hospitals, healthcare providers) or agencies that report fact-checked health information (e.g. WebMD, Healthline); 2) digital media (e.g. news outlets, magazines, and opinion/blog posts); 3) governmental organizations (i.e. funded by federal, state, or local government, such as the Centers for Disease Control and Prevention and public health departments); 4) advocacy organizations (e.g. Truth Initiative, American Heart Association) or professional societies (e.g. American Academy of Family Physicians, American Academy of Pediatrics); 5) academic journals; and 6) other sources. Data from the coders were combined and duplicate webpages eliminated (n=1156). An additional 180 webpages were excluded because their links were no longer active (n=116), a subscription was required (n=1), they had no original content and contained only links to other webpages (n=13), they were product advertisements (n=38), or they contained only videos or images (n=12). The resulting sample consisted of 464 webpages. The average coding concordance across all categories was 91.1% (range: 76.2% for information targeting non-users to 97.4% for source of information). Disagreements were resolved through discussion until consensus was achieved. Finally, the reading grade level of the text of each of the 464 webpages was determined by the Flesch-Kincaid Grade Level, a widely used measure of readability, and was obtained using Web FX (https://www.webfx.com/tools/read-able/). The Flesch-Kincaid Grade Level measures the average number of words per sentence and syllables per word to determine the grade level at which the text can typically be understood^23^. Scores range from 0 to 18, where a score of 0 represents a kindergarten reading level and each additional point up to 12 represents the associated grade level (e.g. 5 indicates a 5th grade reading level). Scores beyond 12 represent collegiate or graduate levels of reading^23^.

### Statistical analysis

As searches were conducted in the US and Google Chrome Incognito is sensitive to location, we assessed readability according to US-based guidelines (i.e. AMA) and calculated the proportion of webpages that met recommended reading levels (i.e. ≤6th grade). Then, independent samples t-tests were used to examine differences in reading level between webpages: 1) targeting teens only vs parents only; 2) targeting e-cigarette users only vs non-users only; and 3) with positive vs negative or neutral messaging. Finally, analysis of variance (ANOVA) was used to compare reading level between webpage sources. For this analysis, webpages published by academic journals were excluded, as they do not target a general audience and would be expected to have a higher reading level. Data were analyzed using SAS version 9.4.

## RESULTS

On average, webpage content was written at approximately a 7th grade reading level (Flesch-Kincaid Grade Level Mean=7.34, SD=2.22), and 23.3% of webpages met the AMA guideline of ≤6th grade reading level (range: 11.5%–45.2%; Table 1). There were no differences in average reading level for webpages targeting teens only compared to those targeting parents only [t(85)= -0.96, p=0.34] or users only compared to non-users only [t(255)= -0.41, p=0.68]. There were also no significant differences in average reading level for webpages with positive compared to negative or neutral vaping messages [t(462)=0.77, p=0.44] or based on the webpage source [F(4, 433)=0.34, p=0.85].

**Table 1:** Proportion of webpages meeting the American Medical Association readability guideline

	Mean (SD)	Meets AMA guideline % (n)
**Total sample** (N=464)	7.34 (2.22)	23.28 (108)
**Target audience** *		
Teens only (n=42)	6.58 (2.71)	45.24 (19)
Parents only (n=45)	7.08 (2.03)	22.22 (10)
Users only (n=186)	7.17 (2.31)	26.34 (49)
Non-users only (n=71)	7.30 (2.06)	22.54 (16)
**Messaging**		
Positive (n=15)	6.91 (1.31)	13.33 (2)
Negative or neutral (n=449)	7.36 (2.24)	23.61 (106)
**Source**		
Educational or scientific institutions (n=96)	7.35 (2.22)	18.75 (18)
Digital media (n=171)	7.12 (2.00)	26.90 (46)
Governmental organizations (n=59)	7.10 (2.24)	30.51 (18)
Advocacy organizations or professional societies (n=47)	7.20 (2.13)	23.41 (11)
Academic journals (n=26)	9.65 (3.79)	11.54 (3)
Other (n=65)	7.36 (1.40)	18.46 (12)

Means (SDs) are based on Flesch-Kincaid Grade Level^23^ scores of webpages. AMA: American Medical Association. AMA guideline is text written at or below a 6th grade reading level^18^.

* Target audience frequencies do not sum to the total sample size because not all webpages had a specified target audience.

## DISCUSSION

Because the internet has become a frequent source of health-related information^13^, the current study sought to assess the readability of online information about e-cigarette cessation. Results indicated that, on average, text was written above the recommended 6th grade reading level and that only 23.3% of webpages adhered to recommendations. Also, there were no significant differences in readability based on target audience, message valence, or source. Overall, it appears that most of the online information on e-cigarette cessation is written at a level that many readers cannot readily comprehend.

These findings generally mirror those of previous readability studies^20,21^. The proportion of webpages that met AMA readability guidelines was similar to the 24% reported by Wodi et al.^20^. Contrary to the Park et al.^21^ findings, there were no significant differences in readability based on message valence. However, the number of webpages promoting vaping in the current study was very small (n=15), likely due to the focus on cessation rather than e-cigarette use more generally. Small sample sizes also may have hindered our ability to detect differences based on target audience, or perhaps cessation-related material tends to be general, rather than designed for specific audiences. It is noteworthy, however, that the category with the highest proportion meeting readability guidelines was webpages targeting teens. Although this finding may indicate that attention is being paid to making content for teens easier to read, recommendations suggest that most youth would benefit from e-cigarette-related information that is written at an even lower reading level than the recommended 6th grade level^20^.

Our findings indicate that online information about e-cigarette cessation is not written at a level that most people would easily understand. Thus, more attention needs to be paid to ensuring text is written at an appropriate level when developing online content. Emphasis should be placed on writing in short sentences, using words with fewer syllables, and avoiding jargon. To achieve these goals, content developers should ensure they are assessing readability as they develop new and revise existing material.

### Limitations

There are several limitations to the current study that warrant consideration. First, only webpages written in English were included in the analyses. Given that 22% of US residents over the age of 5 years do not speak English at home^24^, it will be important for future work to examine the readability of non-English language webpages as well. Second, as noted above, the number of webpages in some categories was small. More research is needed examining whether webpages tailored to specific audiences would be more effective in communicating health-related information. Third, our analysis focused on textual information and did not include visual content, such as images or videos. Further, vaping information targeted to teens using more visually-oriented platforms (e.g. YouTube, TikTok, Instagram) also would be interesting to examine. Future studies should consider investigating visual content as well as the interplay between textual and visual content to understand more fully the quality of health-related information. Finally, the current study examined only readability and did not explore the accuracy of information contained on these webpages. Additionally, future studies would benefit from conducting content analyses to determine whether popular websites are providing accurate information.

## CONCLUSIONS

Despite these limitations, the current study indicates that most web content related to e-cigarette cessation is not written at a level easily understood by general audiences and therefore needs to be written more simply to facilitate comprehension. Thus, health communicators advocating e-cigarette cessation should pay particular attention to the readability of information, especially that conveyed via webpages given the increasing reliance on online content, to ensure wide accessibility of information.

## Data Availability

The data supporting this research are available from the authors on reasonable request.
